# Supplementary material for: Cholesterol Regulates Airway Epithelial Cell Differentiation by Inhibiting p53 Nuclear Translocation
Source: Int J Mol Sci. 2025 Aug 27;26(17):8324. doi: 10.3390/ijms26178324 (PMC12428349; doi:10.3390/ijms26178324)
Supplement: Supplementary file 1 [file ijms-26-08324-s001.zip › ijms-3713063-supplementary.pdf]

Supplementary Materials

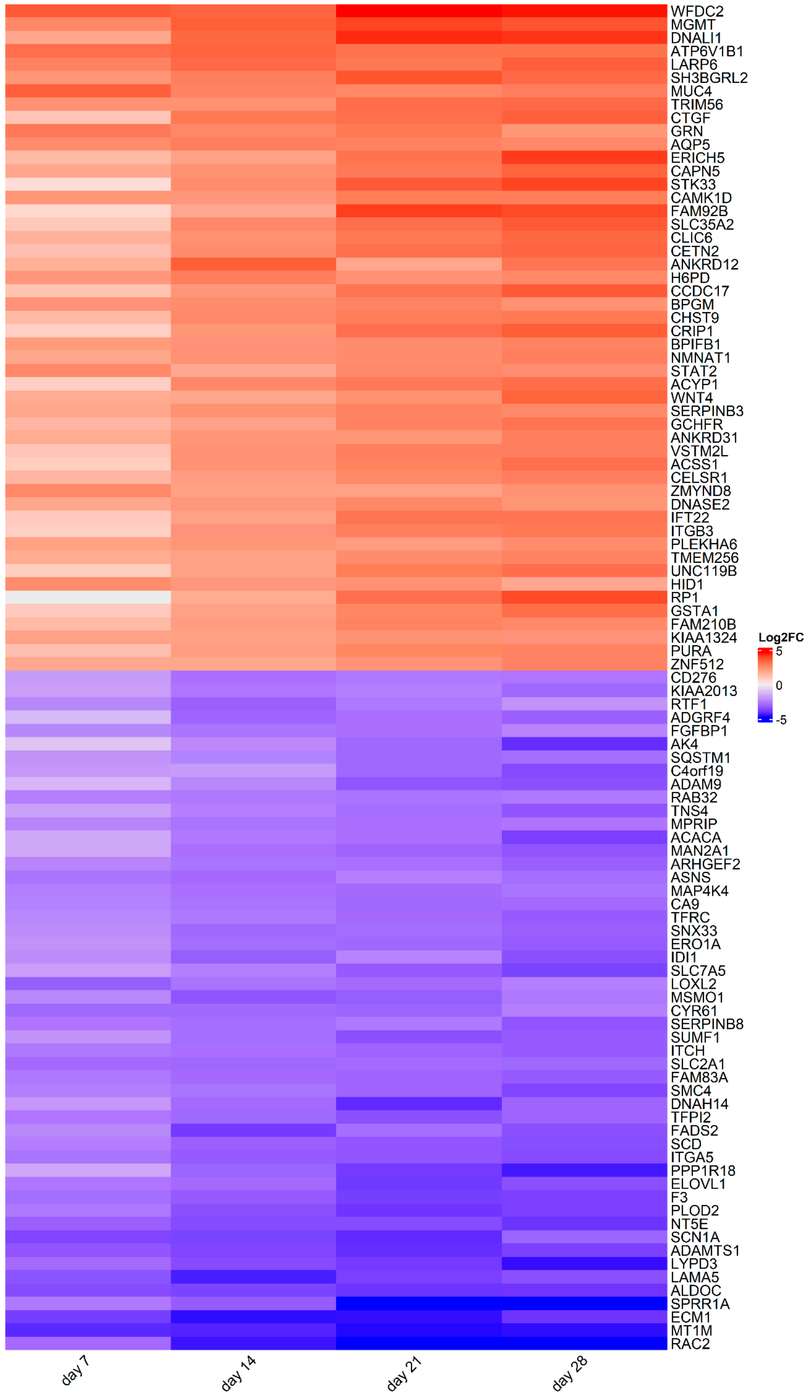

Figure S1. List of top 100 differentially expressed proteins from normal differentiating phBECs. Heatmap containing the list of top 50 up and downregulated differentially expressed proteins during four weeks of normal phBECs differentiation in comparison to day 0. Statistical analysis of normal differentiating phBECs proteome data was performed for four time points (day 7, 14, 21, and 28) as compared to day 0 using Wald test with Storey correction to account for multiple testing to identify differentially expressed proteins (significance value $q < 0.05$).

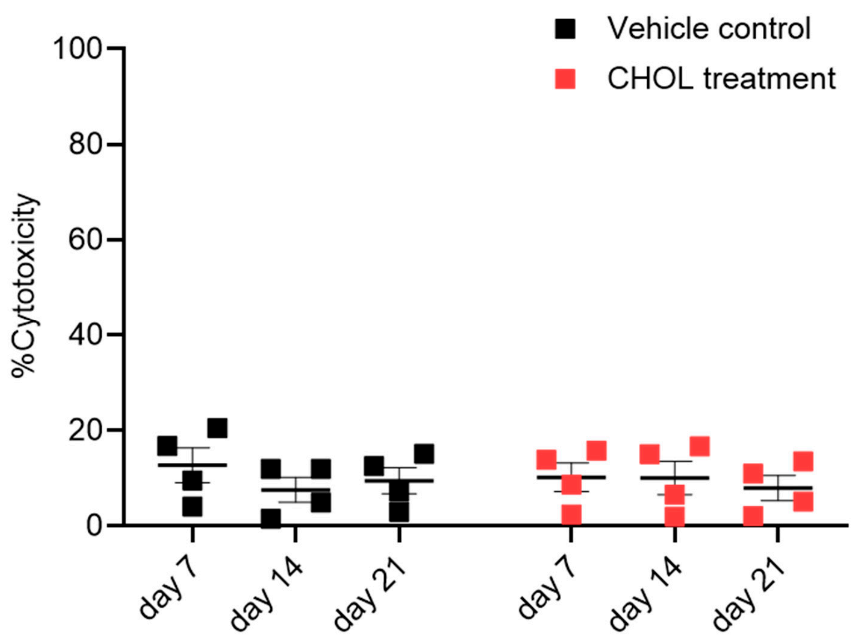

Figure S2. Chronic CHOL treatment does not induce cytotoxicity in differentiating phBECs in vitro. The cytotoxicity assessment was determined using lactate dehydrogenase (LDH) assay after chronic CHOL treatment normalized to the 2% Triton X positive control for the above mentioned timepoints during the differentiation phase. Data presented as mean ± SEM (n = 4). The statistical analysis was performed for chronic CHOL-treated phBECs in comparison to time-matched vehicle-treated phBECs using a two-tailed paired *t*-test with Bonferroni correction to account for multiple testing. No significant changes were observed.
